# Real-Time Compliant Stream Processing Agents for Physical Rehabilitation

**DOI:** 10.3390/s20030746

**Published:** 2020-01-29

**Authors:** Davide Calvaresi, Jean-Paul Calbimonte

**Affiliations:** Institute of Information Systems HES-SO Valais-Wallis, University of Applied Sciences and Arts Western Switzerland HES-SO, TechnoPole 3, CH-3960 Sierre, Switzerland; davide.calvaresi@hevs.ch

**Keywords:** stream reasoning, real-time multi-agents, RDF stream processing, stream processing agents, digital rehabilitation, real-time sensors

## Abstract

Digital rehabilitation is a novel concept that integrates state-of-the-art technologies for motion sensing and monitoring, with personalized patient-centric methodologies emerging from the field of physiotherapy. Thanks to the advances in wearable and portable sensing technologies, it is possible to provide patients with accurate monitoring devices, which simplifies the tracking of performance and effectiveness of physical exercises and treatments. Employing these approaches in everyday practice has enormous potential. Besides facilitating and improving the quality of care provided by physiotherapists, the usage of these technologies also promotes the personalization of treatments, thanks to data analytics and patient profiling (e.g., performance and behavior). However, achieving such goals implies tackling both technical and methodological challenges. In particular, (i) the capability of undertaking autonomous behaviors must comply with strict real-time constraints (e.g., scheduling, communication, and negotiation), (ii) plug-and-play sensors must seamlessly manage data and functional heterogeneity, and finally (iii) multi-device coordination must enable flexible and scalable sensor interactions. Beyond traditional top-down and best-effort solutions, unsuitable for safety-critical scenarios, we propose a novel approach for decentralized real-time compliant semantic agents. In particular, these agents can autonomously coordinate with each other, schedule sensing and data delivery tasks (complying with strict real-time constraints), while relying on ontology-based models to cope with data heterogeneity. Moreover, we present a model that represents sensors as autonomous agents able to schedule tasks and ensure interactions and negotiations compliant with strict timing constraints. Furthermore, to show the feasibility of the proposal, we present a practical study on upper and lower-limb digital rehabilitation scenarios, simulated on the MAXIM-GPRT environment for real-time compliance. Finally, we conduct an extensive evaluation of the implementation of the stream processing multi-agent architecture, which relies on existing RDF stream processing engines.

## 1. Introduction

The demographic changes in our society, including the lengthening of life expectancy, entails several challenges regarding healthcare support and assistance for older adults. Chronic diseases and health issues affect an increasing amount of people among this population, often leading to a decrease in the quality of life. Some of the key factors related to this decline are the diminution of physical activity and the consequent reduction of mobility [1,2]. Many different clinical conditions can be co-factors originating impairment situations. Nevertheless, factors such as falls, progressive loss of mobility, and lack of exercise have also shown to have a high impact [3,4]. Numerous studies have demonstrated the effectiveness of physical therapy for improving life conditions in different situations, including post-operatory interventions for hip replacement, cancer survival treatments, back pain, stroke rehabilitation, among many others [5,6,7]. However, to maximize the efficacy of these rehabilitation strategies, providing personalized treatment, feedback, and support is essential. Moreover, several treatments (especially due to chronic conditions) require continuous monitoring over extended periods, often including home-based exercises and periodic check-ups. Furthermore, given the wide variety of physical rehabilitation procedures with healthcare professionals, patients tend either to abandon the treatments quickly or to decrease the adherence to the proposed exercises progressively.

Digital rehabilitation emerges as a promising approach that consists of leveraging information and communication technologies for boosting the efficacy of physical rehabilitation interventions [8]. These technologies include, among others: (i) the usage of sensing devices with monitoring capabilities (e.g., able to capture motion and physiological data) (ii) applications based on machine learning and other data analytics methods; (iii) the deployment of distributed intelligent systems with streaming capabilities and in-time feedback.

In the last decade, the progress of the Internet of Things (IoT) and wearable sensors has paved the way for the implementation of integrated systems, which produce data in the form of continuous streams that require dynamic processing techniques [9]. In addition to the velocity and volume dimensions related to data streams, the variety of the data produced is particularly relevant when dealing with distributed heterogeneous sensors. This variety has been mitigated through the usage of machine-readable semantic data models (i.e., ontologies and knowledge graphs) in the areas of stream reasoning [10] and RDF stream processing [11]. Nevertheless, in application domains such as physical rehabilitation, in-time feedback is needed, as otherwise, a delay might jeopardize a measurement or endanger the safety of the patient [12]. Therefore, in such cases, traditional streaming data processing algorithms are not sufficient. In these scenarios, to guarantee strict processing deadlines and time restrictions is essential. Although such constraints have been long-studied in the discipline of real-time systems, currently available (semantic) IoT architectures do not provide support for real-time compliant policies and protocols [13].

Recently, real-time techniques have been explored in the context of autonomous intelligent devices and multi-agent systems (MAS), with results that may have a potential impact in digital rehabilitation scenarios [12]. However, these works still have not explored the integration of real-time techniques within semantically-enabled stream processing systems. Thus, to support practical applications demanding for distributed intelligent entities interacting dynamically via the exchange of semantic data streams and negotiating/executing time-critical tasks, this paper addresses the following challenges:

- The inclusion of strict real-time compliance in RDF stream processing systems,
- The combination of autonomous real-time agents and semantic stream processing, and
- The application of real-time stream processing agents in digital physiotherapy scenarios.

With respect to the challenges mentioned above, the contribution of this paper can be summarized as follows:

- The definition of a model for real-time compliant stream processing agents, constrained by strict deadlines for interactions and negotiation among participating agents.
- The study and implementation of agent-based stream processing entities, based on the RDF stream processing paradigm.
- The simulation and analysis of both real-time and general-purpose MAS, considering different scenarios for digital rehabilitation using motion sensors.

These contributions constitute a concrete advancement with respect to the state of the art, especially regarding the inclusion of real-time strict constraints on stream processing agents. In this context, the main objectives of this work are: (i) to formally define real-time constraints in RDF stream processing agents; (ii) to propose an architecture of real-time multi-agent RDF stream processing system; (iii) to show the feasibility of the stream processing agents approach, through a concrete implementation based on existing RDF stream processing engines; and (iv) to demonstrate the effect and consequences of real-time constraints in simulated scenarios of a multi-agent digital rehabilitation application.

The remainder of the paper can be summarized as follows. In Section 2 we describe the field of digital rehabilitation and how multi-agent systems and semantic streaming data have been applied in recent years. In Section 3 we present the main challenges of real-time autonomous systems for digital rehabilitation, followed by the description of our model and architecture in Section 4 and Section 5. Section 6 describes the simulation scenarios and their results, while Section 7 provides details of the implementation and experimentation on our architecture for RDF stream agents. Finally, Section 8 concludes the paper.

## 2. Digital Rehabilitation

Conventional rehabilitation practices are characterized by unilateral, interactive bilateral, and cooperative bilateral [12] interactions. In the framework of such interactions, for either physical or cognitive rehabilitation, the main activities that need to be addressed are training, counseling, monitoring, and assessment. The transition towards digital rehabilitation is mainly driven by the will of fastening the follow-up, enhancing the healing process, shortening the hospitalization, lowering the costs for both patients and health structures, enabling continuous monitoring, providing equitable access to rehabilitation services and finally supporting technological advancements in telemedicine [14]. In recent years, the increasingly broad range of available technologies for physical activity monitoring enabled a face-paced advancement of these approaches. Application domains in the scope of digital rehabilitation include the usage of technologies ranging from: video analysis [15,16], wearable technologies [17], robotics [18,19], distributed sensing [20], and gamification [21,22]. This work focuses on physical rehabilitation. Therefore, the next section dives quickly into wearable-based rehabilitation systems.

### 2.1. Wearable-Based Rehabilitation Systems

Wearable technologies constitute one of the key enablers for digital rehabilitation, and are expected to further provide improvements in both preventive and rehabilitation approaches. Although there are still concerns about the potentially invasive characteristics of wearable-based systems, a recent study (targeting patients in an older adult-care facility) revealed that 93% of the patients accepted body-worn sensor systems [23]. Concerned about the possible reluctance in using wearable-based systems (i.e., stigmatized-like), Bergmann et al. [24] reported a surprisingly positive assessment of the aesthetics of those systems. Nevertheless, major concerns still arise with respect to restricted recording time (e.g., due to limited storage capacity or limited battery-life), wearability, and reliable real-time feedback.

Wearable sensors employed in digital rehabilitation systems can span from being “simple” micro-sensors (e.g., capturing inertial movements or biomedical information by using small, intelligent, and low-energy active devices) to be “complex smart” (e.g., capturing data via thin and flexible sensors, compatible with textiles or made of textile technologies with specific mechanical, electrical or optical properties). Examples of rehabilitation systems based on this type of sensors have been applied to different use cases including exercise assessment in knee osteoarthritis [25], upper-limb motor training for stroke patients [26], or classification of motor activities for COPD patients [27]. In all these systems the focus is on ensuring the accuracy and efficiency of the sensing process and the results they produce in order to address the specific use-case needs. However, in all these systems the autonomy of sensing devices and the compliance to strict time constraints are not considered.

### 2.2. Real-Time Feedback and Agents in Digital Rehabilitation

The multi-agent system (MAS) approach is a comprehensive paradigm for modeling complex (distributed) systems and their dynamics. An agent is a (partially) autonomous entity, embodying a program, a sensor, a robot, or even a human being operating (sensing and actuating) in a given environment.

In the area of digital rehabilitation, we can count a plethora of contributions coming from the MAS community. For example, Rodriguez et al. [28] proposed a system assisting upper limb rehabilitation. Agents in this system perform abstract tasks such as (i) recording the movements while the patient is executing the exercise, (ii) receiving specific inputs (e.g., BPM, skin conductance), and defining the level of stress/fatigue, and (iii) behaving like a “virtual therapist”, adapting the therapy according to the current level of stress of the patient. Felisberto et al. [29] developed a MAS able to recognize patients’ movements and postures and to detect possibly harmful activities. Exploiting a wireless body area network (WBAN) as an underlying system, an intelligent agent analyzes cyclically possible variations in the values received. The ultimate goal is to identify physical/posture deterioration. The consumption of medical drugs is a common eventuality in rehabilitation scenarios. Indeed, Mutingi et al. [30] proposed an agent-based system to cope with decision-making processes in drug delivery.

Summarizing, concerning the “lower” layers of wearable-based systems, they are able to perceive and pre-elaborate in-loco kinematic and biomedical parameters, and no one is powered by MAS. However, if further analysis is required, proprietary (often closed) solutions have to be involved. Conversely, solutions employing MAS can provide sophisticated, extensible, and scalable analysis. However, it has to be highlighted that it is challenging to deploy MAS on wearable sensors. The reasons hampering MAS from pervading wearable and embedded sensors stem from both technical and technological limitations [12]. In particular, MAS lack of technological means (e.g., frameworks) to be seamlessly deployed in distributed/wearable sensors characterized by scarce computational resources. Moreover, from the technical perspective, traditional MAS algorithms are incapable of dealing with strict timing constraints—crucial characteristic to deliver in-time feedback (typical of physical rehabilitation). Indeed, a recent study identified and formalized the challenges for MAS to be compliant with strict-timing constraints [13]. In turn, Calvaresi et al. [12] elaborated on the challenges related to the compliance of MAS with strict deadlines and embedded architectures. Moreover, the authors proposed and detailed a viable solution to bridge the gap between MAS and real-world wearable-based rehabilitation systems. Finally, an ultimate element still needs to be taken into consideration in the overall picture: the capability of processing streaming data in MAS for rehabilitation purposes [9].

### 2.3. Semantic Data Stream Processing

Considering the prominent role of sensors and wearable devices for digital rehabilitation, it becomes a necessity to (i) provide the means for managing data streams from these sensors through rich semantic models, (ii) enable the processing of querying and reasoning of these semantic streams, and (iii) provide real-time mechanisms for decentralized and autonomous interactions among stream processors. A number of RDF Stream Processing (RSP) systems have been developed in the last decade, focusing on the processing aspects of semantic streams, including incremental reasoning, continuous querying, complex event processing, among others [31,32,33,34,35]. However, most of these RDF stream processors rely on centralized architectures for interaction and organization, even if at least in theory they rely on Web standards.

In the literature, early attempts to provide REST-ful service interfaces for streaming data were explored in [36,37]. These exploratory proposals introduced the usage of Web infrastructure, which evolved towards prototype developments such as the RSP Service Interface (http://streamreasoning.org/resources/rsp-services), which further develops the ideas presented in [36], providing a generic implementable programming API for continuous query engines. The distributed execution of RDF stream workflows over a network has further been explored in the SLD Revolution framework [38], which optimizes interactions using lazy-transformation techniques so that non-optimized RDF formats are used only when necessary.

Regarding the publication of streams from distributed producers, TripleWave [39] enables the transformation of non-RDF data streams, as well as time-annotated RDF, into RDF streams. TripleWave allows the publication of these RDF streams so that they can be directly consumed or connected with applications that process them. The concept of TripleWave is further expanded in WeSP (http://w3id.org/wesp/web-data-streams), a conceptual model for producing and consuming RDF streams on the Web.

Even though the notion of decentralization is a fundamental aspect of the Web, in most of the streaming data and processing models the deployment of autonomous stream processors effectively remains challenging. The vision introduced by the Semantic Web [40], and its related initiatives, considered agents as primary actors for the generation and consumption of data on the Web. However, most implementations of the Semantic Web have focused on ontology modeling, reasoning engines, Linked Data, or RDF data querying, but have relegated agents to a marginal position. This also applies to semantic-aware streaming data processing (i.e., RSP and stream reasoning approaches). Although the introduction of agent and multi-agent approaches has been introduced recently [9,41], it is still required to provide concrete models, specifications, and implementations of viable agent-based stream processing and reasoning systems. Finally, regarding the inclusion of real-time constraints for RDF stream processing, so far most approaches have focused on best-effort stream processing, without the notion of compliance to strict deadline constraints.

## 3. Autonomous Real-Time Streaming Agents for Digital Rehabilitation

Digital rehabilitation can be applied to a wide variety of practices. Let us focus on the use case of post-operatory knee physiotherapy. After surgical intervention, and the subsequent hospitalization, patients are traditionally left alone at the time of discharge, with a prescription that includes the series of exercises (therapy) that they are supposed to do. Suche exercises may include simple strength and flexibility conditioning programs, e.g., heel cord stretch, standing quadriceps stretch, or supine hamstring stretch.

Commercial solutions in the market for monitoring this type of exercise have a number of significant limitations, especially regarding precision and usability. For example, devices such as the Kinetec [42] are used for performing passive and continuous knee movements during rehabilitation. This device is typically used during the acute phase, allowing to control among other parameters the angle of the knee movement. However, the angle of the machine does not precisely correspond to the angle of the knee itself, mostly because of structural reasons, limb misplacement, or attempts to compensate for the movement performed by the patient trying to reduce an undetectable pain [12]. This limitation leads to an inadequate assessment of pain, muscular resistance, and evolution of the treatment, added to other potentially misleading information due to the unsupervised usage of the Kinetec device.

**Figure 1:**
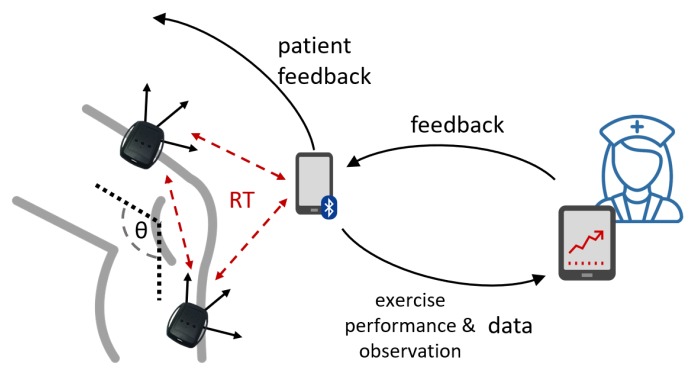
Digital rehabilitation scenario: knee motion sensors for exercise monitoring and real-time feedback. In this scenario the knee sensors interact in real-time with the patient’s application, which in turn provides immediate feedback, and provides information to the health professional about the exercise performance.

As opposed to conventional physiotherapy with episodic encounters with health professionals to keep track of the patient’s progress, the digital rehabilitation approach can rely on sensing devices for accurately capturing the movements of the patient during the exercises. These include triaxial accelerometer measurements, which can be used to calculate the flexion angle of the knee, number of repetitions, coordination of movements, duration of the stretching episodes, etc. However, this scenario requires the sensors to autonomously coordinate and organize their interactions and data exchange within strict time constraints. For instance, two knee sensors computing flexion angles during physical exercises must synchronize their interactions in real-time (Figure 1). If these real-time deadline constraints are violated, the misalignment in the combined measurements jeopardizes the accuracy of the angle calculations, resulting in inaccurate monitoring of the exercise performance. Hence, once the angles have been calculated, the patient may need to get in-time feedback about the exercise (e.g., through visual/audio indicators in her tablet or mobile device). Again, this feedback requires to be bound in time. Otherwise, it would provide entirely useless information to the patient. In particular, in the case of coaching, late and jeopardized feedback can be potentially dangerous: e.g., a late stop feedback can generate an over-extension exceeding the safety range established for that given patient. Finally, the physiotherapist and other physicians (or healthcare providers) may need to monitor the exercise live, potentially on a tele-rehabilitation scenario. This last interaction could require real-time feedback (in case of immediate response from the physiotherapist) or standard communication requirements (in case of asynchronous feedback or off-line monitoring).

**Figure 2:**
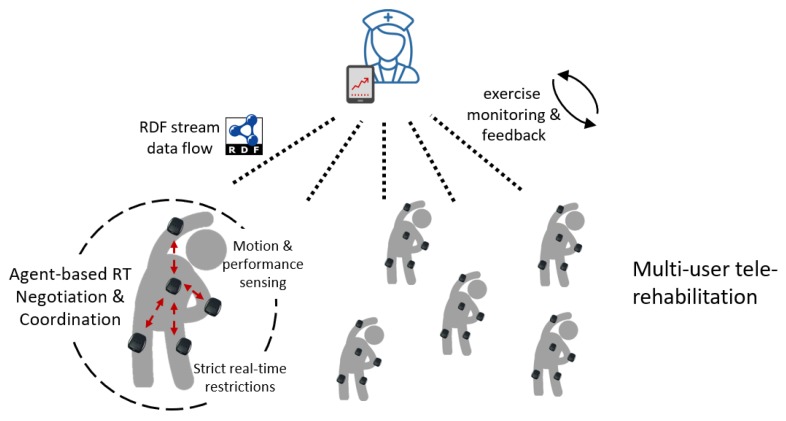
Digital rehabilitation scenario with multiple patients: decentralized and autonomous stream data management for heterogeneous sensors. Expanding from the previous scenario, real-time stream processing agents coordinate to provide per-user monitoring and feedback, while RDF streams are used for semantically-aware data exchange within a group of patients. The healthcare professional can then monitor and provide personalized feedback according to individual performance.

Extending this one-patient scenario to a setting where multiple patients are followed throughout their treatment entails additional challenges (e.g., managing the heterogeneity of the different sensors, increasing the potential activities to be monitored, and scaling to real-time multi-patient digital rehabilitation monitoring). Indeed, each patient might follow a specific therapy, therefore having different treatment prescriptions and body-parts to rehabilitate (thus possibly requiring various sensors—in number and capabilities). Moreover, the evolution of the patient can also be different in terms of compliance, time, and efficacy. For all these reasons, it is essential that beyond the real-time interactions within the sensor environment, a digital rehabilitation system must also rely on semantic models for representing information across patients and healthcare providers, using technology standards such as RDF (Figure 2).

As it has been described in [12], a number of functionalities are needed in this type of digital rehabilitation scenarios, including tools and methodologies for supporting: (i) adherence to the treatment; (ii) monitoring of performance and correctness of the movements; (iii) enabling adjustments, management of errors and compensations; (iv) coaching, encouraging and motivation of the patient; (v) providing motivation, commitment and fatigue measurements; and (vi) incorporating practice-specific parameters. Multi-agent systems have provided relevant contributions to all of them [43], although breaching distributed sensing in real-world applications is still an open challenge. The usage of affordable wearable sensors is essential to allow monitoring and in-time feedback in digital rehabilitation scenarios. Indeed, a previous work [12] elaborated on the employments of MAS for telerehabilitation, proposing a theoretical model and relevant future steps to undertake. Undoubtedly, the success of digital rehabilitation will also require the integration of diverse sensing technologies and semantic compatibility. Although the question of sensor data heterogeneity has been addressed in past years, with promising results regarding the inclusion of ontology-based approaches for stream processing in IoT environments, the integration of these methods with real-time compliant technology remains mostly unexplored. Essential challenges in this respect include the addition of real-time constraints in existing models such as RDF stream processing, or the representation of behaviors and negotiation using semantic technologies.

Having described the context of digital rehabilitation and the need for autonomous systems for managing in-time interactions among sensor devices, we elaborate the following set of challenges, which need to be addressed:

- Autonomous sensor interactions. Beyond traditional IoT deployments configured following top-down paradigms; digital rehabilitation often requires autonomy on the configuration of the devices, as well as their synchronization and negotiation over data and services.
- Real-time guarantees in sensor processing. Given the necessity of complying with deadlines and strict constraints on data execution, negotiation and delivery, autonomous sensors must incorporate scheduling mechanisms to ensure these real-time guarantees.
- Standard and extensible messaging and metadata. Sensing devices should be able to exchange data, in different formats and representations, potentially using Web standards for representing metadata. Possible information to be specified are time constraints, performatives, conditions, and negotiation protocols.
- Asynchronous and distributed communication. Sensors should be able to send and receive messages, as well as coordinating among them without the need of a central entity that governs their interaction flow.
- Semantic stream data management. Semantic representations should be employed to allow sensors to understand and act according to a given stream of data. These representations should align with Web standards (e.g., OWL, RDF), and allow extensibility and high expressiveness.

## 4. Real-Time Stream Processing Agents Model

Having enumerated the main challenges related to real-time compliance in stream processing agents for applications in the digital rehabilitation domain, in this section we introduce an agent-based model that addresses those issues. First, we describe the RDF stream processing formalization used throughout the paper. Then, we propose a multi-agent model in which behaviors are linked to time-bound constraints. Finally, we describe how interactions among these agents are established, using semantic standards.

### 4.1. RSP Data Model

The RSP model describes how data streams can be represented as potentially infinite sequences of RDF triples annotated with timestamps [44]. According to [45], we define an RDF triple as a tuple:

(s,p,o)∈(I∪B)×I×(I∪B∪L),

where s is the subject, p is the predicate, and o is the object; and *I*, *B*, and *L* are the disjoint infinite sets of IRIs, blank nodes, and literals, respectively. A set of RDF triples is called an RDF Graph. Moreover, collections of RDF Graphs can be represented as RDF datasets. A named RDF graph is a pair:

(n,G),withn∈(I∪B),andGisanRDFgraph.

Then, an RDF dataset is a set *D* defined as:

$$D=\{G_{0},\left(n_{1},G_{1}\right),\left(n_{2},G_{2}\right),\ldots ,\left(n_{i},G_{i}\right)\}.$$

The RDF graph $G_{0}$ is called the default graph for *D*, and $\left(n_{j},G_{j}\right)$ are named graphs, with j∈{1,2,…,i}.

These time-agnostic definitions can be extended to support the notion of RDF streams, seen as a sequence of special RDF datasets, or RDF stream elements, each of which is formalized as follows:

$$s=(G_{t},\left(n,G\right)).$$

$G_{t}$ carries the timestamp of the stream element, including a triple of the form (n,p,τ), where *p* is a predicate that describes a timestamp annotation (e.g., schema:observationDate, or ssn:resultTime from well-known ontologies such as SSN, SOSA, or Schema.org), and τ is the timestamp. For simplicity, we represent the timestamp of a stream element as $\tau_{s}$. The named graph *G* contains the payload of the stream element. Then, a stream *S* is defined as an unbounded ordered sequence of stream elements:

$$S=(\ldots ,s_{i},\ldots ,s_{j},\ldots ).$$

In this sequence, for every $s_{i}$ and $s_{j}$, the order is established by their timestamps, i.e., $\tau_{s_{i}}<\tau_{s_{j}}$, where $\tau_{s_{k}}$ is the timestamp in $G_{t}$ of the stream element $s_{k}$. Furthermore, each stream *S* may be identified by an IRI *n*, in a tuple (n,S).

Given the unbounded nature of streams, windows are defined as a way to refer to portions of the stream over time, so that processing and querying operators can be applied. A window *W* applied over a stream *S* consists of a finite set of stream elements from *S*. Different strategies can be used to extract this finite subset of elements from the stream. In this work, we focus on time-based windows, defined as follows:

$$W_{u,v}\left(S\right)=\left\{s|s\in S\;\mathrm{and}\;u\leq \tau_{s}<v\right\}.$$

In this time window, $W_{u,v}$ the time parameters *u*, *v* represent an interval that delimits the contents of the window based on timestamps of the stream elements. Notice that a window length could be specified instead of the interval upper bound. To illustrate the usage of windows, we present an example of a CQELS [33] query (Listing 1) that obtains heartbeat measurements form a specific sensor, under a window of 2 seconds. In this case, the stream is identified through a URI, over which the window boundaries are specified. Notice that different strategies can be applied as to how and when the contents of the window are filled (e.g., when the window closes, or as soon as the window content changes).

**Listing 1.** CQELS continous query over a stream of heartbeat observations.

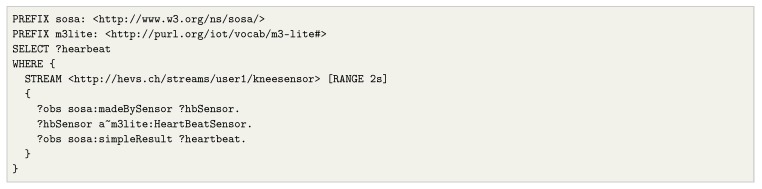



### 4.2. Stream Processing Agent Model

A stream processing agent α can be formalized as a tuple with the following structure:

α=(K,G,B,F),

where *K* represents the agent’s beliefs, *G* corresponds to the goals, *B* represents the behaviors, and *F*, a selection function.

The set of beliefs *K* is represented as RDF statements on a dataset. In the case of data streams, *K* may also include stream elements from one or more streams, available through the application of a time window, as explained above. As an example, consider Listing 2, which represents a sequence of RDF stream elements on a stream in JSON-LD format. This specific example shows heartbeat observations, each of which is contained on a timestamped graph, as indicated in the model introduced previously. The agent may use this information as part of its beliefs, although as the stream is potentially infinite, it may only keep the latest heartbeat values, typically bounded through a time window.

**Listing 2.** RDF stream elements containing heartbeat observations in JSON-LD. This example shows how stream elements can be represented as time-annotated graphs and containing RDF triples that represent the stream contents (e.g., sensor observations).

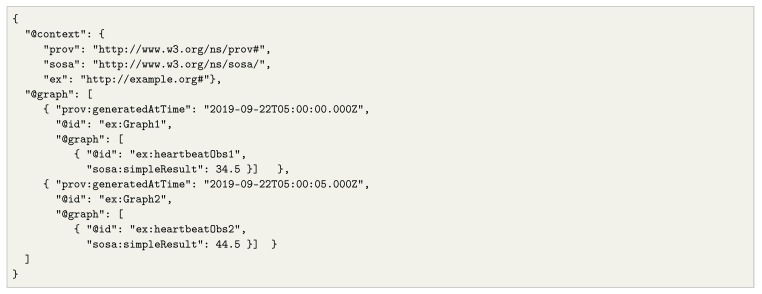



*G* is a set of goals, which account for restrictions and targets defined for the agent. These may include time-bounded restrictions over the desired goals, which may have an impact on the real-time strict scheduling mechanisms, as we will see later.

*B* represents the set of behaviors for the agent. Each behavior b∈B represents a strategy or procedure to solve a certain problem. Each behavior is characterized by the following elements $b=(a,sel_{a})$. a∈A is an action from the set *A* of actions that the agent can execute, while $sel_{a}:K\times \left(D,T\right)\to A$ is the function that defines which action to take depending on the current belief k∈K, and the temporal restrictions: deadline (*D*) and period (*T*). The deadline is the maximum elapsed time to execute the action, while the period is the frequency at which the agent may launch the action.

Finally, *F* is a selection function that specifies which behavior the agent will take, according to the current goals in *G* and beliefs in *K*.

In this paper, for simplicity, we consider the case of simple behaviors, referred to as tasks. One particular task model is named periodic tasks (i.e., tasks whose execution recur endlessly after a fixed period *T*) [46]. Besides the period *T*, and relative deadline *D* this model of task also includes information such as the release time *R*, and computation time *C* (typically a worst-case estimation). To guarantee compliance with strict-timing constraints, the agent local scheduling algorithm evaluates the feasibility of its task-set based on the related utilization factor. In particular, the utilization factor $U_{b}$ of a given task (periodic behavior) *b* in a task-set Γ is computed dividing its computation time $C_{b}$ by its period $T_{b}$ (when period is equal to deadline: T=D). Therefore, the utilization factor of a given agent α at a given time *t* is defined by

(1) $$U^{\alpha }\left(t\right)=\sum_{b\in \Gamma (t)}U\;\;\mathrm{with}\;\;U=\frac{C}{T}.$$

Then, the task-set is considered feasible if its utilization factor is less (or equal) than the least upper bound $\left(\leq U_{lub}\right)$ of its scheduling algorithm [46].

### 4.3. Negotiation among RSP Agents

**Figure 3:**
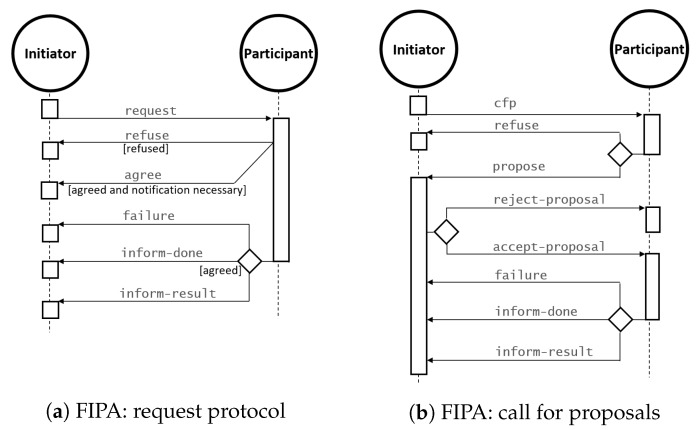
Examples of FIPA interaction protocols (**a**,**b**). The standard protocols establish the way in which agents interact, providing an abstraction of generic message exchanges.

RSP agents interact with each other in a structured manner, through protocols based on existing standards for multi-agent cooperation, negotiation, and delegation. Among these protocols, we focus on those defined by FIPA [47]. In Figure 3 depicts two examples of standard FIPA negotiation protocols. Figure 3a represents a request for a given task/data from a given initiator to a given participant. This latter can accept or refuse the request. If the answer is positive and the initiation awards the task execution, the participant will start performing it.

Figure 3b depicts a protocol in which the initiator launches a call-for-proposals (cfp), asking other agents to bid for the execution of a task. The participant then makes a proposal (or called bid), which can be accepted or rejected by the initiator.

**Figure 4:**
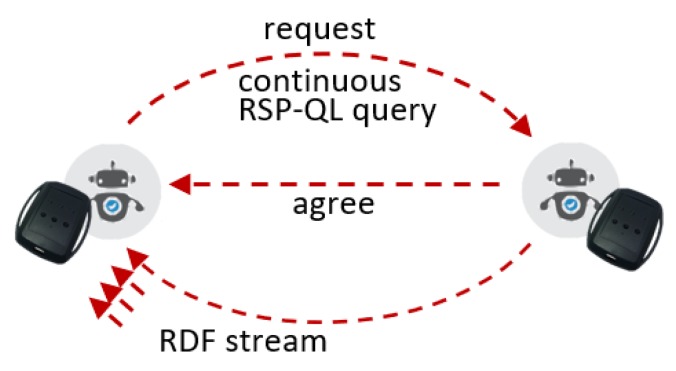
Simple request interaction between two sensor agents: continuous RDF Stream Processing (RSP) query. This interaction can be embedded into a FIPA request protocol, with the addition of a continuous set of final responses instead of a single one. The continuous query will expect multiple responses until the query is canceled or its execution expires.

Using primitive negotiation protocols of this type as building blocks, RSP agents can develop more complex interactions among them. As an example, consider limb sensors producing continuous streams of movement observations in Figure 4. The first agent requires raw data from the second agent and thus performs a request, in the form of an RSP query. After agreeing, the second agent will inform the results of the request, but unlike a traditional request, it will stream the sensor data back. There are different possible ways of actually implementing data delivery, as we will see in Section 5, but in a general sense, we can abstract the stream as a sequence of messages delivered in a specific order.

**Figure 5:**
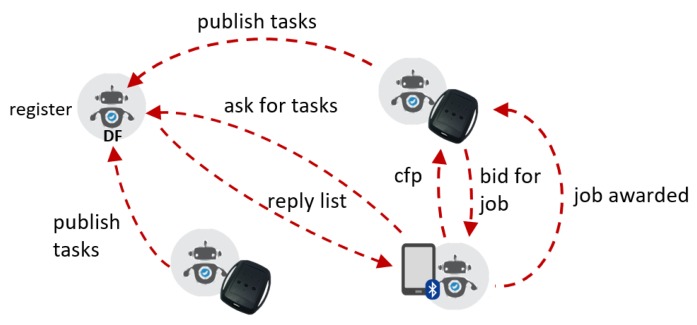
Call-for-proposal interaction among stream processing agents. This interaction can be represented as a FIPA call-for proposals, in which sensor agents publish the tasks they can provide (e.g., sensing and monitoring) in a register agent. Then an application agent can request for agents who are capable of a certain tasks, and emit a cfp. After a bid is performed, the task can be sscheduled and executed.

As another example of interaction among agents in digital physiotherapy in Figure 5, we illustrate a scenario in which the agent organizes and coordinates their work autonomously, on behalf of the sensors and devices they represent. First, two body sensors publish the tasks or behaviors they can provide, using a register agent as an intermediary. These tasks can refer, for example, to the provision of RDF streams of movement computation data, or physiological aggregated data. Then, the agent acting on behalf of the patient monitoring system asks the registry for metadata about those agents providing a specific type of task. Having the list of sensors that provide these features, it emits a cfp request, to which the sensor agents may bid, and get awarded the execution of the job. Once the bid is granted, the exchange of RDF streams can be established using a specific channel (e.g., MQTT, HTTP, and WebSocket).

The protocol described above shows the capacity of RDF stream agents to establish collaboration strategies in a decentralized manner. However, to allow the compatibility and understanding of the protocols, it is essential to represent these interactions through semantic and machine-readable standards. In Listing 3 we show an excerpt of a FIPA call-for-proposals in JSON-LD, which can be used as a message exchanged among RSP agents, e.g., for a scenario such as the one in Figure 5.

**Listing 3.** FIPA call for proposal message represented in JSON-LD. This example shows an excerpt of a message emitted by an agent soliciting a service or task to which other agents may bid for. The message itself is repreented in RDF and is exchanged through the RSP agent interfaces.

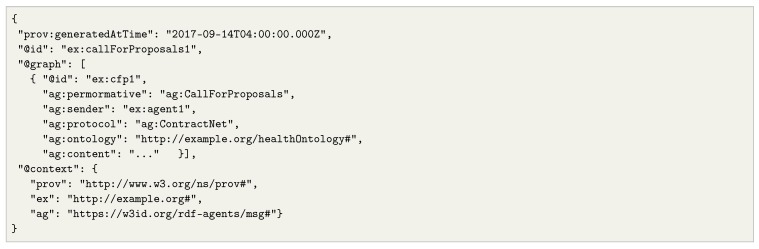



The proposed model presented in this section essentially contributes on three main novel aspects with respect to the state of the art: (i) the addition of real-time constraints in the RDF stream processing model; (ii) the modeling of RDF stream processors (or reasoners) as decentralized agents; and (iii) the alignment of RSP agent interactions as extensions to standard multi-agent system protocols, with a particular accent on sensor-based application such as digital rehabilitation.

## 5. An Agent-Based Architecture for Decentralized RDF Stream Processing

The model described in the previous section introduces the notion of stream processing agents, capable of acting according to beliefs and goals (e.g., knowledge) represented as RDF streams, and including real-time constraints. The streaming nature of the data exchanged by these agents makes it necessary to change certain paradigms regarding the delivery and processing of data, leaning towards continuous data processing mechanisms. The Web provides the necessary infrastructure and standards for enabling decentralized communication and interaction among software agents and constitutes an appropriate foundation layer for the implementation of the model in Section 4. To successfully make RDF streams available on the Web, Dell’Aglio [11] outlined a set of requirements partially derived from the more general guidelines for stream processors [48]. These can be summarized as (1) prioritizing active paradigms for data exchange, (2) combination of streaming and stored data, (3) availability, distribution, and scalability, (4) wide range of stream operations, (5) availability of stream metadata, (6) support for a variety of streams, and (7) reuse of existing protocols and standards.

While we endorse these requirements in general, this paper emphasizes the need for guaranteeing that RSP engines interact with each other in a decentralized manner, following the nature of the Web. This implies a departure from the usual setting in previous RSP approaches, where a server-centric paradigm governs continuous query processing and data flows among entities. An example of such an approach is reflected in the interaction patterns of a continuous query workflow, where the entire focus is solely on the query engine server.

**Figure 6:**
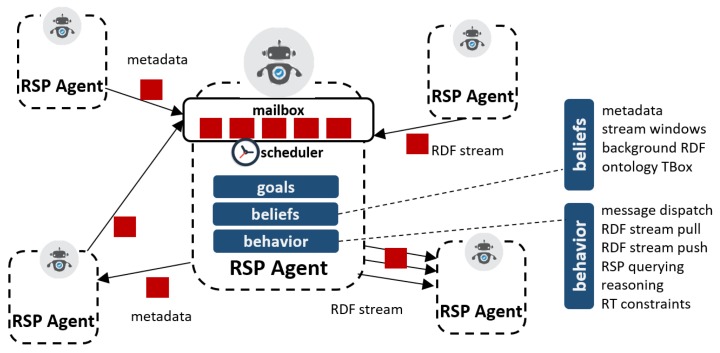
RSP agents architecture. Each agent encapsulates beliefs, goals, and behavior, and manages incoming messages through its mailbox. The execution of real-time constrained tasks is governed by an internal scheduler, and the constraints themselves are included in the agent behavior. Ontologies and vocabularies are included as part of the agent beliefs or knowledge, as well as the contents of dynamic stream that the produce/consume. Interactions among RSP agents happen through RDF message exchange of either metadata or continuous RDF streams.

We propose an architecture centered on RSP agents (i.e., autonomous agents that can be deployed in a distributed fashion and that are able to communicate and exchange RDF streams) and their corresponding metadata (Figure 6) through their inbox.

As described in the model, each RSP agent encapsulates a set of beliefs, goals, and behaviors, and interacts with other agents exchanging messages and streams of data through its inbox. Each agent may act as a sender or receiver of two main types of messages:

- RDF stream elements: these are RDF triples or graphs from a given RDF stream, as defined in Section 4. The stream delivery of these messages, either pulled or pushed, can be applied in different scenarios (e.g., feeding a stream, delivering query answers, and pushing reasoning entailments).
- RDF stream metadata: these are essentially metadata messages required to perform tasks such as retrieving a stream description, declaring and RDF stream, filter a set of stream endpoints, and declaring a query.

Each agent is linked to a unique identifier that can be used to locate it, and it can have a set of endpoints, which can be used to reach the RSP agent resources (i.e., its streams and metadata). The resources of each RSP agent includes the metadata of the RDF streams it manages, as well as other information relative to them (i.e., background RDF datasets, RDF stream buffers, ontology TBoxes, and RDF constraint rules). Not all of these resources need to be accessible to other agents. The goals and beliefs of the agent are typically private and may evolve over time, as they may be affected by changes in incoming RDF streams. The behavior of each agent defines how it proceeds at the arrival of incoming messages. In particular, it typically implements internal processing mechanisms such as continuous query processing, complex event processing, and stream reasoning. To do so, the agent may emit new messages (e.g., response to a query), create new agents (e.g., a pushing emitter, or a subscriber handler), or schedule other actions (see Figure 6).

Regarding the requirements mentioned above, this architecture addresses them in the following ways. For requirement 1, it natively supports asynchronous message passing, including the ability to push streams of messages if necessary. Concerning the combination of streams and stored data, the model takes a no-sharing approach for state information, so, in principle, any stored RDF data is only locally accessible and modifiable. The solely allowed procedure to exchange it is through message delivery, which is fundamental also to guarantee scalability and distribution (requirement 3). The behavior of each RSP agent allows sufficient freedom and flexibility to implement different types of operators and processing mechanisms (requirement 4), while the explicit definition of RDF stream metadata covers (requirement 5). The variety of streams is not restricted by the model (requirement 6), and the usage of well-established standards is also advocated.

Finally, the execution of behaviors is governed by the scheduler component, which may include different strategies [49] depending on the type of tasks and the constraints it may have. In particular, in this paper, we refer to real-time compliance to strict deadlines for task execution, as described in Section 4.

### 5.1. Messages and Notifications in RSP Agents

The RSP agent architecture attributes particular importance to the exchange of messages, as they are the basic way to share information and coordinate interactions. The architecture adopts specific considerations about how the messages (also called notifications) are handled, taking into account the differences in dealing with streaming vs. stored RDF data.

Format and vocabularies. Messages in RSP agents fall under two fundamental categories: RDF stream elements, and RDF stream metadata. In both cases, RDF is the underlying data model used to represent the information that is exchanged, although there are minimal expectations. For RDF stream elements, these are expected to conform to the general RDF stream abstract model, as described by the W3C RSP Community Group (https://www.w3.org/community/rsp/) and as in Section 4. However, this abstract model provides high flexibility concerning the use of a particular vocabulary (e.g., using the SSN ontology for representing sensor streams, the event ontology for streams of events, or the PROV ontology for provenance descriptions). Concerning the metadata, it would be advisable to provide a standard vocabulary for RDF stream descriptions, as proposed in [50], although this goes beyond the scope of this paper.

Message storage. RSP engines are designed in such a way that RDF stream elements flow through them, and produce continuous results. Therefore, the stream is not stored, at least not in the way it is done in a traditional database or data store. Consequently, RDF stream elements are accessed through time windows, as in the RSP model, and older messages are bound to fade as time passes.

Message resolvability. As a consequence of the previous observation, it is hard to allow stream messages to be retrievable after an RSP agent has processed them. This differs from other RDF/Linked Data use cases where data dynamics do not follow a streaming paradigm. As we will see later, this leads to the introduction of input and output RDF streams, which restrict RSP agents to either only write or read from a stream. In any case, resolving a particular stream element is of less importance in the context of RSP, than resolving the current contents of a stream, or a view over a stream.

Push message delivery. While on traditional Web standards, pulling is the primary method for delivering data (e.g., through HTTP GET/POST requests), it is not always the most suitable option for data streams. As described below, our proposed RSP agent architecture provides alternative delivery methods, allowing the usage of WebSocket or HTTP Server-sent-events. The abstract description of the RSP agent leaves the message delivery method open for either of the options.

Querying. Given the ubiquity of query access patterns in RDF stream processing, it would be natural to include explicit interaction specifications for registering standing queries, as well as accessing their results as streaming notifications. This applies not only for window-based continuous queries, but also for Complex Event Processing, and given use cases in stream reasoning.

### 5.2. Stream Receivers, Senders and Consumers

The RSP agent architecture specifies three main types of agents: Stream Receiver, Stream Sender, and Stream Consumer. An RSP agent may play the role of one or all of these types.

#### 5.2.1. Stream Receiver

The Stream Receiver is a profile for an RSP agent that is capable of receiving and processing the following types of messages:

- RetrieveAllStreams: to request metadata of all RDF streams registered in the receiver agent.
- CreateStream: to request the declaration of an RDF stream. This message includes the metadata of the RDF stream to be created.
- RetrieveStream: to request for the metadata of a given RDF stream in the receiver agent. Its IRI identifies the requested stream.
- SendStreamItem: to add a stream element to an existing RDF stream residing in the requested receiver agent. This message includes the stream element itself, as well as the RDF stream IRI.
- RetrieveStreamItem: to request for a specific stream element. The message includes the IRI of the RDF stream and the element itself. As in-stream systems and element might be volatile, in the sense that it might not be de-referenceable after some time, this message also includes views over stream elements (e.g., based on time recency).
- PushStreamItems: to request for stream items to be pushed back. The message includes the RDF stream IRI.
- CreateQuery: to request for a continuous query to be registered. The query includes the reference to the stream IRIs to be used and the IRI of the resulting stream of responses.

The Stream Receiver will act upon arrival of any of the above messages to its inbox. We show in Algorithm 1 a sketch of how the agent reacts to these messages. The receive method of the agent is the interface used to indicate what action to take in each case.
**Algorithm 1** Stream Receiver: receive function.1:**procedure**receive(msg)2:  sender←msg.sender3:**switch**msg:4:**case** RetrieveAllStreams:5:  send(getAllStreams) to sender6:**case** CreateStream:7:  ack←postInputStream(msg.body)8:  send(ack) to sender9:**case** RetrieveStream:10:  send(getStream(msg.uri) to sender11:**case** SendStreamItem:12:  postStreamItem(msg.uri,msg.body)13:**case** RetrieveStreamItem:14:  send(retrieveStreamItem(r.uri)) to sender15:**case** PushStreamItems:16:  handler←pushStreamItems(msg.uri)17:  handler.onReceive(data):18:    send(data) to sender19:**case** CreateQuery:20:  ackgetspostQuery(msg.body)21:  send(ack) to sender

The steps taken are self-explanatory in most cases. The Stream Receiver calls internal methods, for instance to create a stream (postInputStream) or to retrieve a stream item (retrieveStreamItem). In practice, these methods will be implemented on top of exiting RSP, CEP, or stream reasoners, binding their native implementations to this interface.

The Stream Receiver can manage several RDF streams registered within it. Streams may be of two different kinds:

- Input streams: these streams are essentially meant only to receive new items, but are not intended to be consumed by other agents other than the one that hosts it. Examples of such streams are those used as input for RSP queries: other RSP agents can feed these streams, but the query processor on the Stream Receiver is the only one that consumes it.
- Output streams: these are those RDF streams that are meant to be consumed by other RSP agents, but fed only by the agent that hosts it. An example of such a stream is the continuous result of an RSP query engine.

RDF streams can also be available as both input and output.

#### 5.2.2. Stream Sender

This type of RSP agent characterizes those interactions related to sending RDF metadata, as well as RDF stream contents to another agent. The sender defines the following basic operations:

- postStream: send RDF stream metadata to declare it on a Stream Receiver. The sender emits a CreateStream message through this operation.
- postStreamItem: send and RDF stream element to an (input) stream on a given Stream Receiver. This is typically a feed-stream message.
- postQuery: register a query on a Stream Receiver with a CreateQuery message.

Apart from these operations, a sender must also be able to discover the Stream Receiver endpoints. For this, it has a discover operation, which for a given stream IRI, requests the endpoint or endpoints available for sending (or consuming) stream elements. The sender also has operations to retrieve the metadata of a given RDF stream, or all available RDF streams on a receiver (getStream, and getAllStreams, respectively). These operations are common to a Stream Consumer, described below.

#### 5.2.3. Stream Consumer

A Stream Consumer characterizes RSP agent interactions relative to receiving RDF stream data. It essentially defines two operations:

- getStreamItem: requests to consume an RDF stream item. Implementations of this operation can derive different strategies for retrieving RDF stream contents. Given the dynamicity of streams, it is usually unfeasible to collect them one by one through their identifiers. Alternatively, these implementations may instead rely on stream views that may capture, for example, the latest stream items in a given window of time, or the ones complying to some filtering criteria.
- pushStreamItems: requests stream items to be pushed to the consumer. Conversely to the previous operation, which is essentially poll-based, this one requests the receiver to act as a sender as soon as there is an RDF stream element available for consumption.

**Figure 7:**
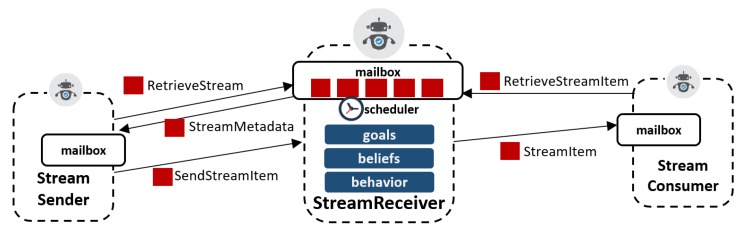
RSP Stream Receiver processes incoming messages from the sender that posts RDF stream items, and sends RDF stream items to a consumer. In case of real-time constraints, the scheduler will rely on a real-time compliant strategy to satisfy the established policies.

For example, let us consider the Stream Receiver depicted in Figure 7. First, it receives a message on its inbox, requesting the metadata of a specific stream. The receiver dispatches back the metadata to the requester, which then post new elements to this stream. Then a consumer may also request a specific stream item to the receiver through a corresponding message. To make these interactions possible, the agents need to first have the addresses of the other agents, and if necessary, discover their endpoint locations.

The RSP agent architecture detailed in this section constitutes a first specification of how the stream-reasoning agent’s vision [9] can be implemented. Beyond existing stream processing engines for RDF data and stream reasoners, which typically work based on top-down organization and communication approaches, this architecture relies on decentralized RSP agents, capable of self-organizing and coordinating processing actions and streaming data exchange.

## 6. Stream Processing Agents Simulation

To design and assess the behavior of a real-time compliant multi-(streaming)-agent system for digital rehabilitation, we have set up a virtual environment using the MAXIM-GPRT [51] simulator. More precisely, the case study presented in Section 3 has been used to define scenarios in which real-time compliance is assessed, under different conditions and parameters. The simulator enables the design and analysis of multi-agent behaviors composed of both General-Purpose (GP) and Real-time (RT) algorithms. Therefore, MAXIM-GPRT has been used to design, simulate, and assess the performance of several agents’ setups with respect to performance metrics such as compliance with strict timing constraints (deadline miss ratio), utilization factors, negotiated workload, response time, and lateness.

### 6.1. Simulated Scenarios and Setups

We studied the following scenarios in the context of digital rehabilitation:

S1: One physiotherapist, one patient, two sensors;
S2: One physiotherapist, two patient, two sensors;
S3: One physiotherapist, one patient, five sensors.

The S1 scenario can refer to a simple rehabilitation setting, in which body sensors capture motion data from a specific part of the body (e.g., knee movement) during particular exercises. The patient monitoring application is also represented through an agent, which can coordinate with the physiotherapist agent. S2 refers to a similar scenario, but extended to more than one patient. The scenario in S3 considers more motion sensors (and agents), for more complex rehabilitation treatments. These scenarios serve as a basis for exploring the potential of the agent-based architecture and allow a system designer to choose the most suitable configuration for a given situation.

These scenarios have been studied considering real-time assumptions for negotiation and interaction among agents. In particular, according to [13], (i) Earliest Deadline First (EDF) has been employed as agent local scheduler (coupled with the Constant Bandwidth Server (CBS) to deal with sporadic and aperiodic tasks) [46]; (ii) it has been assumed a bounded-time delay (e.g., between 10 and 50 ms) communication middleware (RTPS-like) [52,53]; and (iii) the Reservation-Based Negotiation protocol (RBN) has been used to enforce a real-time compliant task negotiation among the agents [54]. Moreover, to analyze and prove the unsuitability of best-effort approaches (e.g., general-purpose such as FIFO and RR-like) in the studied scenarios, S3 has been studied only varying the agent local scheduler (i.e., FIFO) and the negotiation protocol (i.e., contract Net (CNET) [55]) keeping unaltered inputs and communication middleware. Hereafter, we refer to S3 as S3rt (real-time configuration) and S3gp (general-purpose configuration).

**Figure 8:**
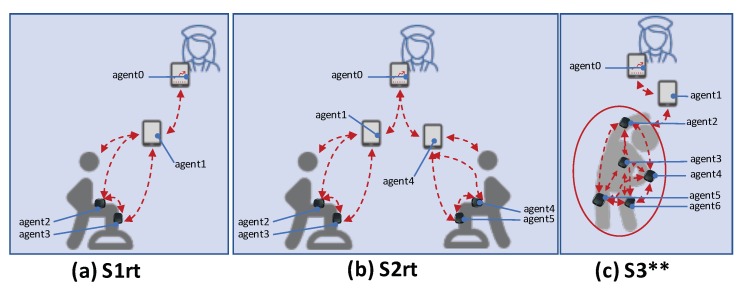
Simulated configurations for S1, S2 and S3. S1 represents a single patient multi-agent scenario, S2 a simultaneous two-patient scenario, and S3 a multi-agent scenario with multiple coordinating body-sensors.

For the sake of clarity, MAXIM-GPRT allows several more real-time and general-purpose configurations. However, assessing the impact of every available algorithm with respect to this case study goes beyond the scope of this paper. The configuration mentioned above produced sufficient results to identify and explain well the challenges, capabilities, and performances of both general-purpose and real-time algorithms at the service of streaming-agents for digital rehabilitation purposes. Table 1 details the general setups of the tested scenarios and Figure 8 shows the role of every agent per scenario.

In this study, we have used simple behaviors, which for simplicity are referred to as tasks. In particular, we have employed both periodic tasks (recurring after a constant period) and aperiodic/sporadic task models (the arrival of the task is not predictable) [46]. Every periodic task is characterized by a task id, the agent executor (Ex), the agent demander (Dm), the worst case of their computational time (*C*), the release time (*R*), a period (*T*), a relative deadline (*D*), first activation (f.R.), last activation (l.R), a flag indicating if that task is public (Pub)—meaning that the agent is willing to perform it on demand, and a flag indicating if such a task is just part of the agent’s knowledge or if it part of its running task-set. Moreover, if the task is aperiodic, *T* cannot be applied. Nevertheless, it can be indicated, if any, a number of executions (*n*) and an associated server to bound its execution [46]. In particular, Task id, Ex, Dm, and *S* are integers IDs; *C* is measured in CPU-clock cycles—normalized in instances of time measured seconds [46]; and R, T, f.R, and *l.R* are instances of time—expressed in seconds.

To enable the agents to demand a task execution, we implemented the concept of need. Such a need is characterized by id of the agent willing to require for its execution, a need id, a release time (*R*), the duration of the bidding window to negotiate its execution (*W*), a starting time to begin the task execution (RT) and its finishing time (TD), a number (*n*) of executions (if not periodic), and a max (maxT) and min (minT) period to execute the demanded task (used only in specific conditions specified at negotiation time). In particular, Need id, Agent id, and task(s) are integer IDs; and R, W, TR, TD, MinT, and MaxT are expressed in seconds. Although the configurations and the workloads have been changed among the scenarios studied, the semantics of the task has been kept the same (see Table 2). The needs generation depends on the specific application scenario (e.g., the need for a specific inertial information given a particular rehabilitating joint.

Fostering fairness, the server handling common aperiodic communication tasks (e.g., read and write messages) have been kept uniform for all the agents among all the scenarios, and are characterized as shown in Table 3.

#### 6.1.1. Scenario S1

As shown in Figure 8a, the mapping agent device is the following: agent 0 → physiotherapist device, agent 1 → patient device, agent 2 → femur sensor, agent 3 → tibia sensor. In this scenario, the kernel tasks have been setup uniform among the devices of the same type. For example, the kernel tasks τ0 are the same for both physiotherapist and patient device, and the same among femur and tibia sensor. The complete characterization of the task-sets employed in this scenario is detailed in Table 4. Table 5 details the needs used to generated the dynamics represented in Figure 8a. In particular, given the agents and task distribution of this scenario, agent 0 needs the information from the participant (in this case, the data are made available by the agent 1, the agent executing on the tablet/smartphone of the only participating patient). In turn, agent 1 needs the inertial information from the wearable sensors (to compute the aggregated plots)—thus it will ask them to agent 2 and agent 3.

It is worth to recall that a task-set $\Gamma^{J}$ of a given agent *j* is feasible if its utilization factor is less (or equal) than the least upper bound (For example, in the case of algorithms such as EDF and CBS $U_{lub}=1$.) $\left(\leq U_{lub}\right)$ of its scheduling algorithm [46]. The utilization factor $U_{k}$ of a single task $\tau_{k}$ is computed dividing its computation time $C_{k}$ by its period $T_{k}$ (in the case where the period $T_{k}$ and deadline $D_{k}$ are equal.): $U_{k}=\frac{C_{k}}{T_{k}}$. Therefore, the utilization factor of a given agent $a_{j}$ at a given time *t* is defined by

(2) $$U^{j}\left(t\right)=\sum_{\tau_{k}\in \Gamma^{j}\left(t\right)}U_{k}.$$

**Figure 9:**
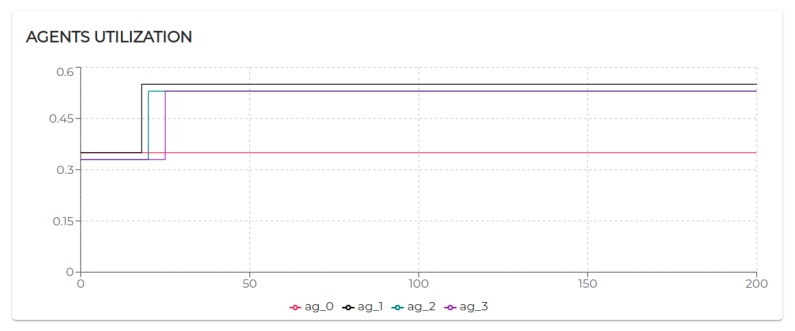
Agens utilization over the simulated time [0–200 s] in scenario S1. y-axis: utilization (adimensional); x-axis: time (seconds).

Figure 9 shows the trend of the utilization factors of all the agents taking part in S1. It is possible to notice that the utilization of each agent does not exceed the upper bound ($U_{lub}\leq 1$) defined for the tested scheduling algorithm (EDF).

**Figure 10:**
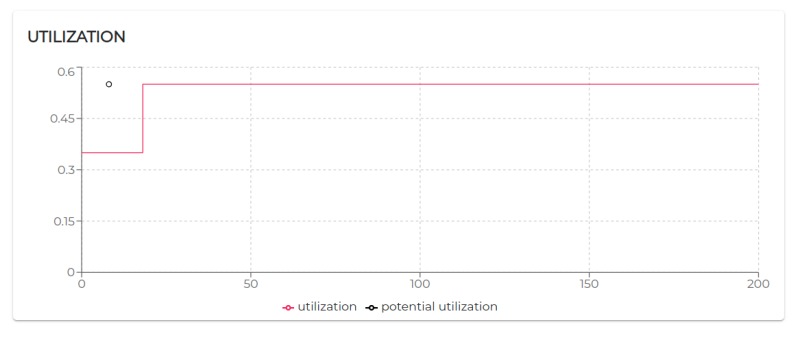
Utilization factor of agent 2 [0–200 s] in scenario S1; y-axis: utilization (adimensional); x-axis: time (seconds).

All the negotiated needs (see Table 5) have been accepted. Hence, as visible in Figure 9, agent 2, agent 3, and agent 4 increase their utilization *U* at a certain point. In particular, looking at Figure 10, it is possible to see that the basic utilization of agent 2 is $U=U_{\tau 1}+U_{S100}+U_{S200}=\left(3/20\right)+\left(1/10\right)+\left(1/10\right)=0.35$. At t=8.06 s, agent 2 negotiates the execution of the task τ5. According to the RBN protocol, the acceptance of a new task is subject to the schedulability test [49]. In this case, adding the τ5 to its task-set would bring its potential utilization factor to $U_{pot}=0.55$ (see black circle in Figure 12). The negotiation ends with agent 0 awarding the execution of τ5 to agent 1. When agent 1 receives such a communication turns its $U_{pot}$ in actual *U* (see t=18.06 s in Figure 10).

**Figure 11:**
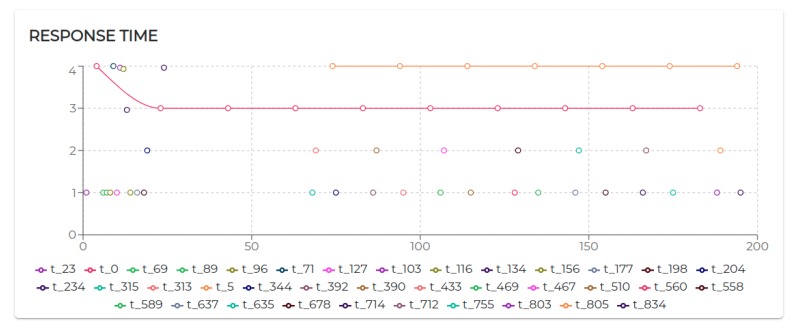
Response time of tasks performed by agent 2 in scenario S1; y-axis: response time (seconds); x-axis: time (seconds).

Figure 11 shows the response time for all the tasks executed by agent 2. It is possible to notice that the design tested in this scenario generates linear response time with negligible exceptions due to a few moments of intense message exchange.

**Figure 12:**
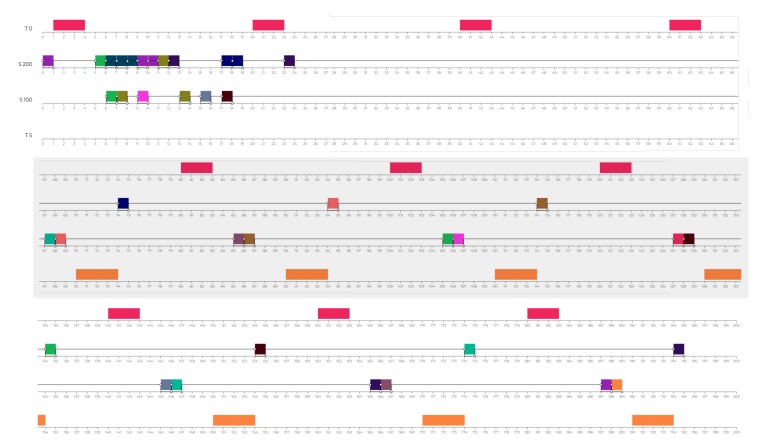
Graphical representation of the local scheduler of agent 2 in scenario S1, [0–200 s]. Due to the length of the selected period, the timeline is depicted in three lines. The task τ5 (in orange) timely displays information after the inertial positions were reported from the agent sensors.

Moreover, starting from t=67 s (see Figure 12), it is possible to see the timely execution of τ1 (twice, reading the inertial positions shared by agent 2 and agent 3) and τ5 plotting those information on the screen of the used device.

#### 6.1.2. Scenario S2

To implement the scenario shown in Figure 8b, the mapping agent device is the following: agent 0 → physiotherapist device, agent 1 → patient 1 device, agent 2 → femur sensor (patient 1), agent 3 → tibia sensor (patient 1), agent 4 → patient 2 device, agent 5 → femur sensor (patient 2), and agent 6 → tibia sensor (patient 2).

To replicate the same conditions per patient, even in S2, the kernel tasks have been setup uniform among the devices of the same type (see Table 6). The complete characterization of the task-sets employed in this scenario is detailed in Table 6. Table 7 details the needs used to generated the dynamics represented in Figure 8b. In particular, in this scenario, we have two patients (with two sensors and one tablet/smartphone each) and one physiotherapist. Therefore, agent 0 will demand aggregated information to agents 1 and 4, which, in turn, will demand inertial information to respectively agents 2 and 3 and agents 4 and 5.

**Figure 13:**
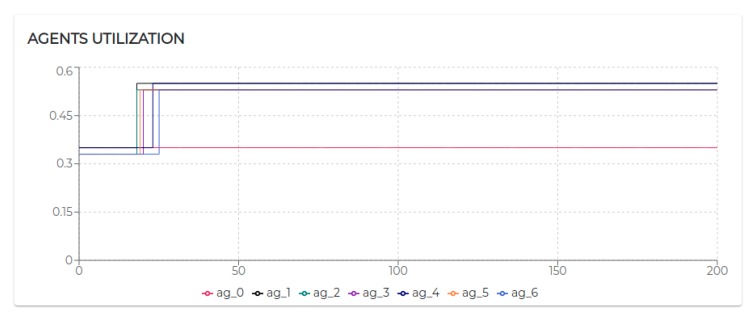
Agents utilization over the simulated time [0–200 s] in scenario S2; y-axis: utilization (adimensional); x-axis: time (seconds).

The Scenario S2 is based on S1, doubling the number of participants (and therefore the number of sensors). On the one hand, the behaviors characterizing patient 1 and his/her device and sensors (agent 1, agent 2, and agent 3) and patient 2 and his/her device and sensors (agent 4, agent 5, and agent 6) remained unaltered. On the other hand, the sole actor potentially affected by the growth of patients is the physiotherapist, therefore agent 0 (Figure 13).

**Figure 14:**
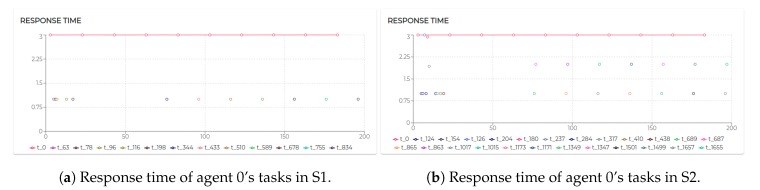
Response times in scenarios S1 (**a**) and S2 (**b**); y-axis: response time (seconds); x-axis: time (seconds).

**Figure 15:**
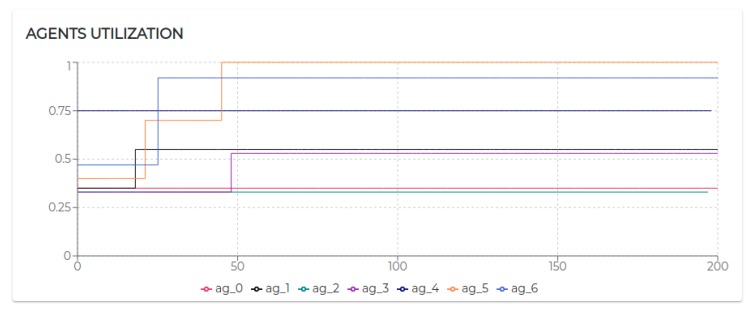
Agents Utilization over the simulated time [0–200 s] in scenario S3rt; y-axis: utilization (adimensional); x-axis: time (seconds).

Hence, although increasing the number of patients does not impact on the response time of the kernel task of agent 0 (see Figure 14b) the response time to process the incoming messages has already been affected, in some cases doubling its value. Figure 15a shows the response time of the agent 0 in S1 and Figure 14b shows its response time in S2. While agent 0 still respects strict timing constraints, the demand for more stringent performance may, at a certain point, require us to adapt the system design to the scale of the application domain.

#### 6.1.3. Scenario S3rt

To simulate behaviors and conditions represented in Figure 8c, the mapping agent device is the following: agent 0 → physiotherapist device, agent 1 → patient device, agent 2 → right arm sensor, agent 3 → chest sensor, agent 4 → left arm sensor, agent 5 → right femur sensor, and agent 6 → left femur sensor. To simulate sensor heterogeneity, in S3rt, it has been assumed different workloads for the several kernel and specific tasks. The complete characterization of the task-sets employed in this scenario is detailed in Table 8. Table 9 details the needs used to generate the dynamics represented in Figure 8c. In particular, agent 0 demands the usual aggregated data for the only participant (agent 1), which, in turn, demands inertial information to all the distributed wearable sensors to compute the complex kinematics of the motor tasks targeted in this scenario.

The execution of the tasks and needs listed above produces the utilization factors plotted in Figure 15. As we can see, beside agent 0 and agent 1 which remained unaltered, the dynamics generated by the release of the needs affected the rest of the agents (especially given their higher utilization factors).

**Figure 16:**
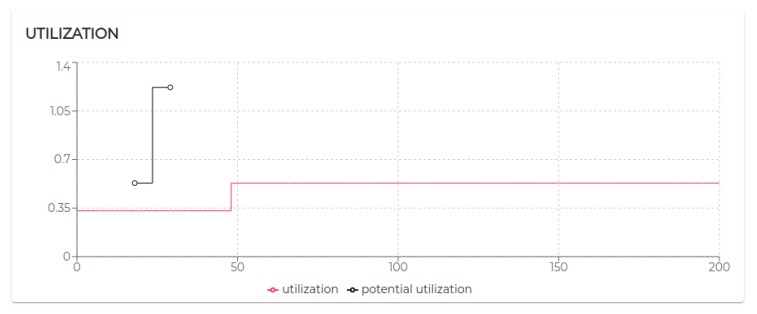
Agent 3 Utilization over the simulated time [0–200 s] in scenario S3rt; y-axis: utilization (adimensional); x-axis: time (seconds).

Indeed, in S3rt, only part of the need has been satisfied. In particular, agent 1 and agent 5 recorded 100% of acceptance and agent 3 and agent 6 accepted only 50% of the demanded tasks. To understand such behavior, let us look at Figure 16. Agent 3 has positively answered (bidded) to the execution of a task, which would bring its utilization to U=0.53. Before the confirmation (award) of such a bid, agent 3 receives a second request. According to the RBN protocol, an agent can accept the execution of a given task only if it can allocate it (without overcoming its maximum utilization factor). In this case, the schedulability test performed at t=29.06 s to verify the possible allocation of the second negotiated tasks brings its utilization at U=1.22. Therefore, the bid for such a task has been negative (rejected). In turn, at t=48 s, the first bid has been awarded. Thus, the potential utilization factor turns into effective utilization.

Finally, let us analyze agent 5 to see how it is performing with the utilization is U=1, the theoretical maximum to still ensure predictability.

**Figure 17:**
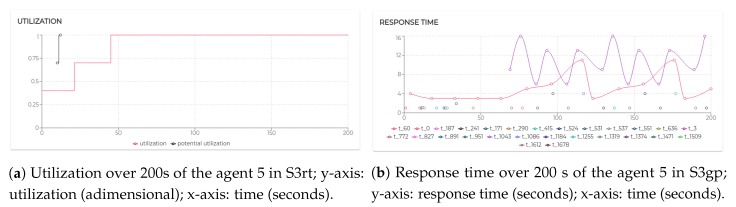
Utilization and response time of agent 5 in S3rt.

Initially, its utilization is U=0.4 (due to running τ0, τ1, and τ2). At t=10.07 s it receives a first request for the execution of τ3, which, if accepted, would raise its utilization to U=0.7. Right after, at t=12.07 s, it receives a second request for executing τ3. At t=12.07 s, agent 5 has not received yet an answer (award/rejection) for the previous bid. Thus, besides its actual utilization is still U=0.4, it has to consider its potential utilization $U_{pot}=0.7$ to perform the schedulability test. Bidding positively to both the requests would bring its potential utilization to $U_{pot}=1$. So, it bids positively once again. At t=21.12 s and t=45.01 s, it gets respectively awarded its two bids. Therefore, as visible in Figure 17a its $U_{pot}$ turns into *U*.

Although operating on the edge of its capabilities, agent 5 does not record any deadline miss during the entire simulation. Nevertheless, high variability in the response time should be acknowledged (see Figure 17b). To refine the response time, adjustments on the initial design might be required. The MAXIM-GPRT tool can be a valuable support in such a process.

#### 6.1.4. Scenario S3gp

In the scenario S3gr, it has been used the same task-set of used in S3rt (see Table 8) and the same needs distribution and characterization (see Table 9).

**Figure 18:**
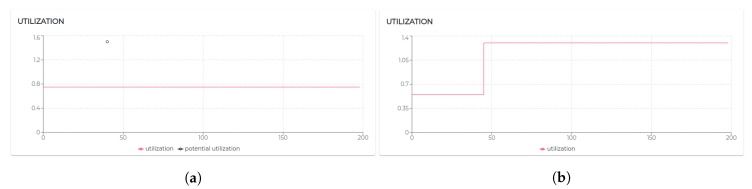
Comparison among the response time over 200 s of agent 4 in S3rt (**a**) and S3gp (**b**); y-axes: utilization (adimensional); x-axes: time (seconds).

However, considering the general-purpose nature of the underlying mechanisms (FIFO as agent local scheduler and CNET as negotiation protocol), no timing guarantee con be enforced nor predicted [13,49,54]. Hence, over 200 s of simulation, agent 3 misses 8% and agent 4 misses 52% of their deadlines. Figure 18 shows that agent 3 at t=40 s refuses to execute a task that would raise its utilization at U=1.5 (which would have entailed unpredictable consequences).

**Figure 19:**
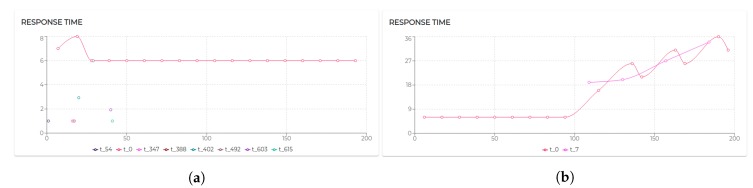
Comparison among the response time over 200 s of agent 4 in S3rt (**a**) and S3gp (**b**); y-axes: response time (seconds); x-axes: time (seconds).

The combination of FIFO and CNET lacks mechanisms typical of real-time systems crucial to handle workloads, deadlines, and strict timing constraints. Hence, in S3gp, agent 4 accepts to execute the demanded tasks. As a result, both the tasks executed by agent 4 record the deadline miss. Clearly, in scenario S3rt, agent 4 has a more conservative (refusing to execute the demanded task), Conversely, in S3gp agent 4 is more flexible (accepting the demanded task), which, however, resulted in compromising its predictability and reliability. To better understand the visibly different response time provided by agent 4 in the two tested scenarios, Figure 19 shows the performances recorded.

**Figure 20:**
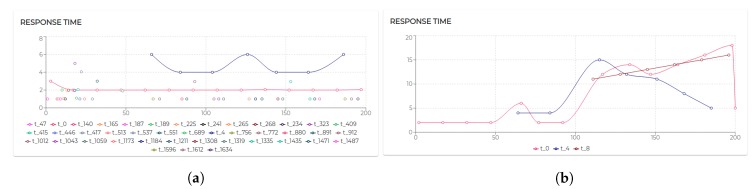
Comparison among the response time over 200 s of agent 4 in S3rt (**a**) and S3gp (**b**); y-axes: response time (seconds); x-axes: time (seconds).

Although less, the flexibility of the general-purpose algorithms cost to the agent 3 to record 8% of deadline miss. The comparison of the response time among scenario S3rt and S3gp is shown in Figure 20.

## 7. Implementation and Experimentation with RSP Agents

We have implemented the RSP agent architecture as a library available in Scala. The code is open-source, and it is available in Github (https://github.com/jpcik/ldn-streams). The core of the RSP agents implementation is written using the Akka Actors library (http://akka.io). Akka provides the essential programming abstractions to create actors, providing message dispatching, remoting, actor hierarchies, and other features. The RSP agent’s implementation defines traits (analogous to interfaces in Java and other languages) for its main types of objects. For instance, the StreamReceiver trait implements the receiver agent described in Section 5.2.1. These traits are independent of the communication layer, i.e., it allows plugging different types of channel implementations, such as MQTT or WebSocket. Additional modules can be plugged into the architecture (e.g., for concrete implementations of specific RSP engines). In our initial implementation, we have focused on using CQELS as underlying RDF stream processors, although we have also implemented classes for C-SPARQL and TrOWL. To allow the integration with these existing engines, it suffices that they provide a JVM-compatible API. RSP agent traits make use of abstract methods that need to be implemented for any specific extension. For example, the StreamReceiver trait defines abstract methods that allow: feeding an RDF stream with graph (consumeGraph), register a query (query), push data results (push), and terminate push and clean resources (terminatePush).

In the remainder of this section, we present a set of experimental results of the implementation of RSP agents. The goal of these experiments is to show how the agent architecture implemented in the library performs under different configurations. By changing the number of senders/receivers, concurrent operations, rates of streaming data flow, etc., we present various scenarios which could be implemented in a digital physiotherapy use-cases. Although in single patient scenarios, the number of senders and receivers would be usually low, when dealing with larger numbers of simultaneous users, the number of required agents will also increase.

In the following experiments, the main metric is the throughput, measured in terms of efficiency (i.e., the rate between the actual number of RDF stream elements processed per unit of time, and the maximum ideal number of processed elements). The choice of this metric is based first on the need to assess the behavior of the platform to different conditions of the input streams (e.g., number of streams/senders, the velocity of the streams, number of parallel processors). The usage of a rate indicator is due to the fact that an absolute throughput is clearly variable depending on the input stream characteristics. Therefore the efficiency rate provides a normalized parameter. Queries and data have been adapted from SRBench [56], using an upgraded version of the datasets, using the new version of the SSN Ontology (https://www.w3.org/TR/vocab-ssn/), and a synthetic generation stream feeder. The original data consists of sensor observations extracted from the LinkedSensorData [57] initiative, based on observations collected since 2002 by 20K sensor stations. There are typically five sensors per station, i.e., a total of around 100,000 sensors in the data set. The sensors measure phenomena such as temperature, position, visibility, pressure, etc. Irrespective of the application domain, the experiments described in this section show the feasibility of the stream reasoning agents architecture in a concrete implementation All experiments were run on Ubuntu 16.04 LTS, Intel Core i7-7700U (3.60 GHz, 8 MB cache, Quad-Core).

**Figure 21:**
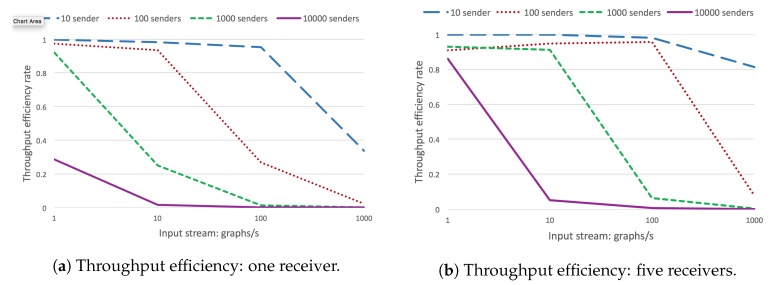
Throughput efficiency vs. input stream rates, for different sets of concurrent senders.

In the first set of experiments, we measured the throughput efficiency, for different input stream rates (1, 10, 10, and 1000 graphs/s), and a different number of concurrent senders, and a single receiver (Figure 21a).

Clearly, the efficiency decreases considerably as the input stream increases. A drastic increase produces a significant drop in efficiency, either if it is by increasing the number of senders or the input rate. This is basically due to the limitations of CQELS as the underlying engine. The next experiment is set in exactly the same conditions, except that it uses five concurrent stream receiver agents instead of only one (Figure 21b). As can be seen, using more receiver agents already provides a higher throughput efficiency for a larger number of cases.

**Figure 22:**
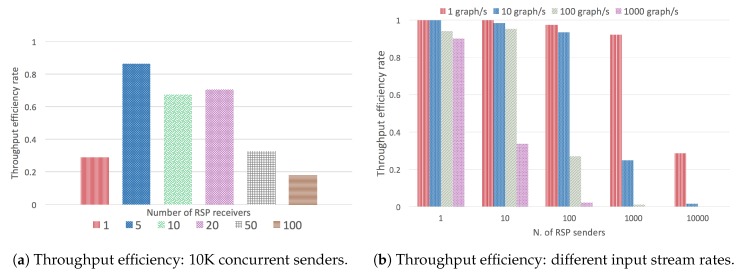
Throughput efficiency vs. number of RSP senders.

The next experiment provides more information on how a set of CQELS engines running as RSP agents can handle a total of 10K concurrent senders, each spitting one graph per second. The experiment is set for 1, 5, 10, 20, 50, and 100 concurrent CQELS agent receivers. As can be seen in Figure 22a, with 5, 10, and 20 concurrent senders, there is a considerable improvement in the throughput efficiency. However, increasing even more receivers produces a sustained decrease, as the CPU is not able to scale on its own to that number of engines. A distributed deployment would be required to scale in that case.

The next experiment shows how RSP stream receivers (CQELS engine) respond to a different number of senders (1, 10, 100, 1000, 1000). It shows results for input streams of 1, 10, 100, 1000 graphs/s (Figure 22b). The graph shows the progressive efficiency decrease as the input stream (combined with the number of senders) increases. For instance, for 10,000 senders, and 1000 graphs/s, the input load is of 10 million graphs/s, which is too much for a single receiver instance.

**Figure 23:**
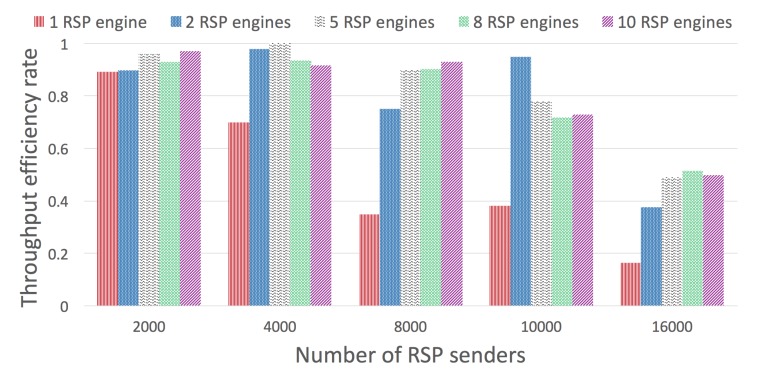
Throughput efficiency vs. number of RSP senders, for different numbers of RSP receiver engines.

In the next experiment (Figure 23), we evaluate a similar scenario, but this time, adding CQELS RSP instances (1, 2, 5, 8, and 10 concurrent engines). The results are shown for different numbers of RSP concurrent senders (2000, 4000, 8000, 10,000, and 16,000). For very high input loads, the system is still capable of at least 0.5 efficiency. It is clear that at this point, a cluster deployment would be required.

**Figure 24:**
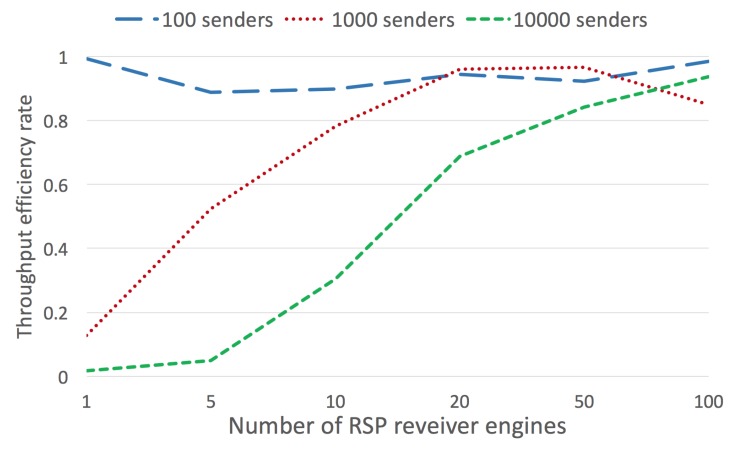
Throughput efficiency vs. number of RSP receivers without processing, for different sets of concurrent senders.

The final experiment was performed only for RSP agents ingesting but not processing data. It shows results for a different number of RSP agents ingesting streams (1, 5, 10, 20, 50, and 100 concurrent agents), and for 100, 1000, and 10,000 senders. As can be seen, for 1000 and 10,000 added additional resources in general increases the overall efficiency (Figure 24).

## 8. Discussion and Conclusions

This paper proposes a novel approach for enabling decentralized stream processing in digital rehabilitation by proposing a stream processing agent-based architecture that enforces real-time constraints. The idea behind these decentralized stream processing agents is that they are capable of sharing not only streaming data, but also processing duties, using collaboration and negotiation protocols, while relying on common vocabularies/ontologies that consider the high dynamicity of their beliefs, state, goals, and behavior [9,58]. These features are essential to provide highly responsive feedback and accurate data analytics, which are required in digital physiotherapy.

Compared to the state of the art, this work provides the following contributions:

- Stream reasoning agents model. Beyond existing approaches in stream reasoning or RDF stream processing (as seen in Section 2), the proposed model does not only focus on complex processing algorithms and methods over semantic streams, but also on the autonomous cooperation among agents that produce and consume those streams, following the vision described in [9].
- Real-time compliance for RSP agents. Existing RSP systems provide execution models that do not support mechanisms for compliance with strict real-time constraints. Filling this gap, our proposed model incorporates these constraints at its core, which can be implemented using existing strategies as in [12].
- Real-time agent simulation results for digital rehabilitation. The simulation environment presented in the paper constitutes an important milestone for modeling and configuring agent-based systems for different scenarios, considering strict real-time specifications. While previous works on digital rehabilitation feedback were typically provided on best-effort strategies, these simulations provide an indication of how and when real-time scheduling strategies can be helpful in order to deal with strict timing limitations.
- Implementation and evaluation of RSP agents. The feasibility and behavior of the RSP agents concept has been demonstrated in this work, through a concrete implementation that relies on an existing RSP engine. This is a first implementation of the agent model for RDF stream processing, beyond the centralized systems present in the literature, as seen in Section 2.

The model, simulation, and implementation presented in this paper also constitute an important milestone towards the adoption of agent-based technologies for real-time sensing applications. The results of this research work open several opportunities, even if there are some limitations that we need to consider, as explained below.

Opportunities. Based on the principles of stream reasoning and RDF stream processing, the proposed model incorporates the flexibility of autonomous organization of streaming agents, with the capability of defining strict deadlines for agent behaviors. This feature is a fundamental advantage for digital physiotherapy, as it enables reliable in-time feedback among sensors and/or eHealth applications for patient support. Moreover, sharing common ontology models and underlying RT-MAS mechanisms, additional sensors and devices can be easily plugged. The approach proposed in this paper relies upon and extends previous works on stream reasoning, also including the representation of heterogeneous data streams as dynamic knowledge graphs on which complex-event processing (CEP) and inductive/deductive reasoning can be applied. This feature addresses the challenges related to sensor and agent heterogeneity, relying on standards for representing agent negotiation protocols and sensor metadata. The simulation results developed in this paper provide an indication that this approach can have a deep impact on digital rehabilitation scenarios, allowing the self-configuration of decentralized sensor solutions. Complementary to these results, the evaluation performed on the RDF stream processing agent implementation, provides evidence of the feasibility of allowing agent-based interactions among sensing devices. Finally, the simulator employed in this study might be a strategic tool for future system design and setup, before including the human in the loop.

Limitations. The simulation scenarios tested in this study have relied on synthetic data generated by the execution environment. In real-world applications, getting such information might require further tasks such as a more complex signal processing and more complex agent interactions (depending on the kinematic chain of a given motor exercise), which might entail more complex semantic representations of the exchanged information. Moreover, many real sensors are still unable to “run” RT-agents (due to the lack of a proper RT-MAS framework for embedded systems). Operating in safety-critical conditions, the development of 3rd-party hardware and software might require a longer developing time, thus slowing down the adoption of the proposed solution. Another important aspect to consider refers to the fact that in this work we do not handle the uncertainty of both streaming and static knowledge. Agent’s beliefs may have different levels of uncertainty, for which techniques such as fuzzy multi-criteria decision-making [59]. Moreover, RSP agents may require to adopt strategies for scheduling streaming task under uncertainty conditions [60], or rely on discrepancy measures in case of disagreements [61]. In terms of impact, this approach may constitute a first step towards a more decentralized understanding of how IoT devices can be used for supporting eHealth applications. Particularly in digital physiotherapy, it would be important to explore the challenges of deploying stream processing agents in clinical environments. Furthermore, it will be crucial to study how real-time constraints might be included as extensions of RDF validation languages such as SHACL [62]. 

## Figures and Tables

**Table 1:** General setup of the scenarios.

Scenario	N. of Agents	Comm. Delay (milliseconds)	Sim. Time (seconds)	DF Scheduler	DF Server	Agent Local Scheduler	Agent Server	Negotiation Protocol	Contractor Heuristic	Award Heuristic
S1	4	10∼50	200	EDF	CBS	EDF	CBS	RBN	ALL	BEST
S2	7	10∼50	200	EDF	CBS	EDF	CBS	RBN	ALL	BEST
S3rt	7	10∼50	200	EDF	CBS	EDF	CBS	RBN	ALL	BEST
S3gp	7	10∼50	200	FIFO	-	FIFO	-	CNET	ALL	BEST

**Table 2:** Task descriptions.

Task	Behavior
τ0	Kernel task
τ1	read message
τ2	write message
τ3	compute inertial information
τ4	compute inertial information
τ5	display graphical information
τ6	synchronization task
τ7	on-board data elaboration
τ8	MIDI signal reproduction

**Table 3:** Common server characterization.

Server ID	Agent	Budget	Period	Type	Task(s) Served
S100	all	1	10	CBS	τ1
S200	all	1	10	CBS	τ2

**Table 4:** Agents’ task-set for Scenario S1.

Agent ID	Task ID	Ex	Dm	C	R	T	D	n	f.R	l.R	S	Pub	Act
0	0	0	0	3	0	20	20	−1	-	-	-	**✗**	**✓**
	1	0	0	1	-	-	-	-	-	-	S100	**✗**	**✓**
	2	0	0	1	-	-	-	-	-	-	S200	**✗**	**✓**
1	0	1	1	3	0	20	20	-	-	-	-	**✗**	**✓**
	1	1	1	1	-	-	-	-	-	-	S100	**✗**	**✓**
	2	1	1	1	-	-	-	-	-	-	S200	**✗**	**✓**
	5	1	-	4	-	20	20	-	-	-	-	**✓**	**✗**
2	0	2	2	2	0	15	15	-	-	-	-	**✗**	**✓**
	1	2	2	1	-	-	-	-	-	-	S100	**✗**	**✓**
	2	2	2	1	-	-	-	-	-	-	S200	**✗**	**✓**
	3	2	-	4	-	20	20	-	-	-	-	**✓**	**✗**
3	0	3	3	2	0	15	15	-	-	-	-	**✗**	**✓**
	1	3	3	1	-	-	-	-	-	-	S100	**✗**	**✓**
	2	3	3	1	-	-	-	-	-	-	S200	**✗**	**✓**
	4	3	-	4	-	20	20	-	-	-	-	**✓**	**✗**

**Table 5:** Agents’ needs for Scenario S1.

Agent ID	Need ID	R	W	TR	TD	n	MinT	MaxT	Task(s)
0	0	4	10	70	200	-	20	25	5
1	0	5	10	60	200	-	20	20	3
	1	5	10	60	200	-	20	20	4

**Table 6:** Agents’ task-set for Scenario S2.

Agent ID	Task ID	Ex	Dm	C	R	T	D	n	f.R	l.R	S	Pub	Act
	0	0	0	3	0	20	20	−1	-	-	-	**✗**	**✓**
	1	0	0	1	-	-	-	-	-	-	S100	**✗**	**✓**
	2	0	0	1	-	-	-	-	-	-	S200	**✗**	**✓**
1	0	1	1	3	0	20	20	-	-	-	-	**✗**	**✓**
	1	1	1	1	-	-	-	-	-	-	S100	**✗**	**✓**
	2	1	1	1	-	-	-	-	-	-	S200	**✗**	**✓**
	5	1	-	4	-	20	20	-	-	-	-	**✓**	**✗**
2	0	2	2	2	0	15	15	-	-	-	-	**✗**	**✓**
	1	2	2	1	-	-	-	-	-	-	S100	**✗**	**✓**
	2	2	2	1	-	-	-	-	-	-	S200	**✗**	**✓**
	3	2	-	4	-	20	20	-	-	-	-	**✓**	**✗**
3	0	3	3	2	0	15	15	-	-	-	-	**✗**	**✓**
	1	3	3	1	-	-	-	-	-	-	S100	**✗**	**✓**
	2	3	3	1	-	-	-	-	-	-	S200	**✗**	**✓**
	4	3	-	4	-	20	20	-	-	-	-	**✓**	**✗**
4	0	4	4	3	0	20	20	-	-	-	-	**✗**	**✓**
	1	4	4	1	-	-	-	-	-	-	S100	**✗**	**✓**
	2	4	4	1	-	-	-	-	-	-	S200	**✗**	**✓**
	6	4	-	4	-	20	20	-	-	-	-	**✓**	**✗**
5	0	5	5	2	0	15	15	-	-	-	-	**✗**	**✓**
	1	5	5	1	-	-	-	-	-	-	S100	**✗**	**✓**
	2	5	5	1	-	-	-	-	-	-	S200	**✗**	**✓**
	7	5	-	4	-	20	20	-	-	-	-	**✓**	**✗**
6	0	6	6	2	0	15	15	-	-	-	-	**✗**	**✓**
	1	6	6	1	-	-	-	-	-	-	S100	**✗**	**✓**
	2	6	6	1	-	-	-	-	-	-	S200	**✗**	**✓**
	8	6	-	4	-	20	20	-	-	-	-	**✓**	**✗**

**Table 7:** Agents’ needs for Scenario S2.

Agent ID	Need ID	R	W	TR	TD	n	MinT	MaxT	Task(s)
0	0	4	10	70	200	-	20	25	5
	1	4	10	70	200	-	20	25	6
1	0	5	10	60	200	-	20	20	3
	1	5	10	60	200	-	20	20	4
4	0	5	10	60	200	-	20	25	7
	1	5	10	60	200	-	20	25	8

**Table 8:** Agents’ task-set for Scenario S3rt.

Agent ID	Task ID	Ex	Dm	C	R	T	D	n	f.R	l.R	S	Pub	Act
0	0	0	0	3	0	20	20	−1	-	-	-	**✗**	**✓**
	1	0	0	1	-	-	-	-	-	-	S100	**✗**	**✓**
	2	0	0	1	-	-	-	-	-	-	S200	**✗**	**✓**
1	0	1	1	3	0	20	20	-	-	-	-	**✗**	**✓**
	1	1	1	1	-	-	-	-	-	-	S100	**✗**	**✓**
	2	1	1	1	-	-	-	-	-	-	S200	**✗**	**✓**
	5	1	-	4	-	20	20	-	-	-	-	**✓**	**✗**
2	0	2	2	2	0	15	15	-	-	-	-	**✗**	**✓**
	1	2	2	1	-	-	-	-	-	-	S100	**✗**	**✓**
	2	2	2	1	-	-	-	-	-	-	S200	**✗**	**✓**
	3	2	-	4	-	20	20	-	-	-	-	**✓**	**✗**
3	0	3	3	2	0	15	15	-	-	-	-	**✗**	**✓**
	1	3	3	1	-	-	-	-	-	-	S100	**✗**	**✓**
	2	3	3	1	-	-	-	-	-	-	S200	**✗**	**✓**
	4	3	-	4	-	20	20	-	-	-	-	**✓**	**✗**
	8	3	-	11	-	16	16	-	-	-	-	**✓**	**✗**
4	0	4	4	6	0	11	11	-	-	-	-	**✗**	**✓**
	1	4	4	1	-	-	-	-	-	-	S100	**✗**	**✓**
	2	4	4	1	-	-	-	-	-	-	S200	**✗**	**✓**
	7	4	-	15	-	16	16	-	-	-	-	**✓**	**✗**
5	0	5	5	3	0	15	15	-	-	-	-	**✗**	**✓**
	1	5	5	1	-	-	-	-	-	-	S100	**✗**	**✓**
	2	5	5	1	-	-	-	-	-	-	S200	**✗**	**✓**
	3	5	-	6	-	23	23	-	-	-	-	**✓**	**✗**
6	0	6	6	4	0	15	15	-	-	-	-	**✗**	**✓**
	1	6	6	1	-	-	-	-	-	-	S100	**✗**	**✓**
	2	6	6	1	-	-	-	-	-	-	S200	**✗**	**✓**
	6	6	-	9	-	20	20	-	-	-	-	**✓**	**✗**

**Table 9:** Agents’ needs for Scenario S3rt.

Agent ID	Need ID	R	W	TR	TD	n	MinT	MaxT	Task(s)
0	0	4	10	70	200	-	20	25	5
1	0	5	35	60	200	-	20	20	3
	1	5	35	60	200	-	20	25	4
3	0	6	10	60	200	-	20	20	3
	1	10	10	80	200	-	20	20	6
4	0	15	10	80	200	-	20	25	6
5	0	25	10	100	200	-	16	16	8
6	0	35	10	90	200	-	20	20	7
